# Experiences and Expressions of Stress Among US Food Industry Workers During the 2020 Covid‐19 Pandemic

**DOI:** 10.1002/ajhb.70338

**Published:** 2026-10-19

**Authors:** Elizabeth Koselka

**Affiliations:** ^1^ School of Public Health Department of Health Systems and Population Health University of Washington Seattle Washington USA; ^2^ Health Systems Research VA Puget Sound Health Care System Seattle Washington USA

**Keywords:** Covid‐19, essential workers, food industry, occupational safety and health, stress

## Abstract

**Introduction:**

The Coronavirus pandemic (Covid‐19) exposed legacies of deep inequality, including who could afford protection from exposure. As part of the essential workforce, food industry workers continued fully in‐person work throughout the pandemic and associated shutdowns.

**Objective:**

To qualitatively examine US food worker experiences and stressors during the 2020 Covid‐19 pandemic.

**Methods:**

Thirty‐four open‐ended phone interviews were conducted from May through September 2020 with people who grew (*n* = 2), processed (*n* = 5), cooked (*n* = 9), sold (*n* = 17), and/or delivered (*n* = 5) food in the United States. Audio recordings were transcribed and coded for themes of stress, health, and workplace environment.

**Results:**

Interviewees reported widespread disruption across US food system workplaces (e.g., reduced staffing, role changes, and increased workloads). Expressions of stress clustered into 4 themes: financial strain, concern about getting/transmitting Covid‐19, sustained uncertainty, and overburdening. Workers' experiences revealed a need for self‐reliance while their work and life environments were repeatedly overhauled.

**Conclusion:**

Results align with similar investigations into food workers' stress and limited protections before, during, and after the pandemic. The study contributes evidence for stronger worker support systems and positions food workers' lived experiences as vital to research and policy.

## 1 Introduction

From March 19 to April 7, 2020, a series of State Executive orders went into effect “that restricted work and movement to only “essential duties” (The Council of State Governments 2020). These extraordinary measures curtailed Covid‐19 outbreaks at the pandemic's onset in the United States (Lyu and Wehby 2020). With their enactment came a new designation in our workforce: essential workers—people who needed to keep coming in‐person to work so the public could access food, medicine, and emergency services (Pritzker 2020). Many of the lowest‐paying job sectors were deemed essential under emergency orders, as were all jobs relevant to sustaining our food system (Koenig et al. 2016; Bateman 2020; The Council of State Governments 2020). These include grocery stores, restaurants, farming and food manufacturing industries (e.g., meat and produce packing plants), food delivery and transportation.

Public alarm and attention during pandemic‐related shutdowns highlighted risks incurred by people who worked in food‐related industries like grocery stores, e.g., low wages, limited benefits, and heightened environmental exposure (Bhattarai 2020; Dorn et al. 2020; Kinder 2020; Sperling 2020), risks that have persisted with little scrutiny or reform (Gottlieb et al. 2025; Minkoff‐Zern and Mares 2025; Moore and Walle 2025). Research examining the pandemic's impacts on food industry workers demonstrates enduring adverse effects, including increased stress and food insecurity, poor mental health, insufficient wages, and heightened employment precarity—all of which disproportionately burden marginalized race/ethnicity and gender identities (Lippert et al. 2021; Loustaunau et al. 2021; Mayer et al. 2022; Austin and Donley 2023; Spence et al. 2024).

To extend literature on biosocial legacies of Covid‐19, this qualitative study examined how people employed in the US food industry described their experiences and stress while working through pandemic conditions in 2020. The study integrates interview narratives with emerging research to show how crisis conditions amplify the structural precarity of food work and erode workers' wellbeing.

## 2 Materials and Methods

This project used open‐ended interviews to collect first‐hand accounts from workers in the food industry about their experiences working through the Covid‐19 pandemic in 2020. Upon approval by the Northwestern University IRB, recruitment began using a combination of flyers and postings on Facebook and Craigslist. Potential participants completed an online screening questionnaire to determine their job sector, title, and location. The questionnaire also included optional demographic questions. Phone interviews lasting about 1 h were completed between May and September 2020. Questions covered work environment, income, schedule, safety precautions and company protocols, then turned to focus on major impacts of the pandemic, including on stress and wellbeing. Interviewees received a Visa e‐gift card in thanks for their time, and audio recordings of the interviews were transcribed and coded using an iterative process in ATLAS.ti Scientific Software Development Gmbh (2020). Qualitative analysis focused on how interviewees expressed stress, a prominent theme threaded throughout questions and conversations.

## 3 Results

Sample characteristics for the study are described in Table 1. Two people grew food (farming, cultivation), five processed it (at processing or packaging plants), nine cooked or served food at restaurants, 17 sold food at grocery stores, and five delivered either meals or groceries in the United States. Although the sample group covered 10 states across the continental US, most interviewees lived in the Midwest region. Fifteen interviewees (44% of the sample) worked multiple jobs, and mean employment time in the food industry was 2 years (ranging 2 months to 11 years). Twelve interviewees identified as women, 22 as men, and their ages ranged from 18 to 54 years, although most were between 25 and 34 years old. Approximately half of participants who reported their race/ethnicity identified as White (*n* = 14). Sample members also identified as Black or African American (*n* = 12), Hispanic/Latino/a (*n* = 4), Asian (*n* = 1), Middle Eastern or North African (*n* = 1), and/or another race/ethnicity (*n* = 1).

**TABLE 1:** Study sample characteristics.*

	*N*
Job sector	
Grocery store	17
Restaurant	9
Food processing	5
Food delivery	5
Farming	2
Location	
Midwest (IL, WI, IN, OH)	25
Northeast (NY, MA, CT)	6
South & West (MS, TX, CO)	3
Time at job	
< 1 year	6
1–2 years	5
2–4 years	8
4+ years	3
> 1 job	15
Age	
18–24	9
25–34	8
35–44	8
45+	2
Female	12
Male	22
Race/ethnicity	
Asian	1
Black or African American	12
Hispanic/Latino/a	4
Middle Eastern or North African	1
White	14
Other race/ethnicity	1

* All 34 interviewees reported job sector and location at screening and could optionally share information regarding time at job, number of jobs, age, gender, and race/ethnicity. Sample sizes vary accordingly.

### 3.1 Expressions of Stress

Interviewees were reeling from repeated overhauls to their workplace and duties, describing inconsistency in hours, pay, and workload since the pandemic began. As they talked through their experiences, expressions of stress took shape most prominently across 4 themes: financial strain, concern about getting or passing Covid‐19, sustained uncertainty, and overburdening. Themes and corresponding exemplar quotes are reported in Table 2.

**TABLE 2:** Selections from interviewees about stress, organized by 4 themes:

Financial insecurity	“I’m like, what the hell? This job and its minuscule pay, compared to the rest of the world—that's considered essential. And that's when we don’t have a pandemic. It's like, why am I saving the world?” *Grocery store worker, MA, interviewed May 28, 2020* “I have a kid to feed so I have to try to figure out the money that I would be getting. I can’t quit—how would I feed my family? That's the thing: I'm kind of in a bind and I'm the provider. When you're the parent of a little kid, you’ve got to take the risk. That's what it's all about: I was gonna provide, and there's really no two ways about it.” *Grocery store worker, IL, interviewed June 4, 2020* “The good thing is they can’t offer us hazard pay, but they’re allowing us to do overtime.” *Food processing specialist, IL, interviewed May 26, 2020*
Concern over getting themself or loved ones sick	“My risk of catching the Coronavirus is extremely high because of the high turnover. I know for a fact (we weren't supposed to know or discuss this) that two people already caught it at my job, on our first shift. 60 people quit the next day. So now we’re down to half‐staff, and that's where the overtime came in. ‘Hey, can you work? Can you do 10‐12‐hour days?’ Being an essential worker is dangerous and very stressful at the same time. That's why it's called essential, right? Because we're feeding the planet.” *Food manufacturing specialist, IL, interviewed May 26* “It's not just about me and the people shopping in the stores, it's when I come home what happens. I’m more concerned when I go home. Could I be carrying something? Did I bring something home? We’re not gonna know – everything takes two weeks.” *Grocery store worker, MA, interviewed May 28, 2020* “When they did call me back [to work], all these feelings just overwhelmed me: ‘I’m going to die. I’ll be dead in a week’…I had to call twice the hotlines where they gave me emotional help, because I was so stressed out I got my kids sick. That's my worst fear. I don’t care if I die. I don’t want to see them die.” *Grocery store worker, IL, Interviewed July 20, 2020*
Uncertainty and instability	“End of March, we hit a brick wall, like whoa: we have to be careful with our money. What are we going to do? I'm freaking out, sending out my resume, feeling really frantic about finding a new income…It was a cascade effect with stress, anxiety and then feeling worthless because I didn't get my nursing degree. I’m going, ‘Oh my god, I can't ask for help now.’ That really shattered my mental health/stability at the moment, but now I'm back on track.” *Grocery delivery driver, IL, Interviewed June 2, 2020* “I’m probably going to have to close down my storefront permanently. I’m 99.9% sure…I think I might be less stressed than before the shutdown, before even knowing what the future would hold, because I don’t have to deal with the store anymore…I’m trying to roll with things; gotta get through it.” *Restaurant chef & owner, NY, interviewed May 27, 2020*
Overburdening	“So, I’m trying to stay safe, trying to get customers what they want. And it's really stressful for me, because I have to work double my number of shifts, since most of colleagues were put out of the job. So, I had to replace them: I have to do my own job, and I do other people's jobs. So, this pandemic has really been stressful for me, I must say.” *Grocery store worker, NY, interviewed Sept. 1, 2020* “It is stressful because you’ve got to deal with people for eight hours, then you’ve got to go home and you might have personal problems. At home, you might get a phone call that stressed you out, you know, somebody's dead or somebody's sick. So, you’ve always got to deal with people. Being an essential worker is stressful because you deal with a lot of people.” *Grocery store worker, IL, interviewed June 30, 2020* “Sometimes I would hear my phone vibrating—the kids didn't know what to do for their homework, or the teacher was saying they’re going to take points off. And it was really horrible.” *Grocery store worker, IL, July 20, 2020*

#### 3.1.1 Financial Strain

Across varying workplace roles and personal situations, interviewees consistently stressed that they needed income, so they needed to come to work, despite additional workplace hazards. As a grocery worker in IL put it, “When you're the parent, you've got to take the risk… I was gonna provide, and there's really no two ways about it.”

#### 3.1.2 Concern About Getting and Transmitting Covid‐19

Interviewees named close quarters, high turnover, long shifts, and insufficient personal‐protective equipment (PPE) as their top concerns. Those in customer‐facing positions like restaurants and grocery stores also worried about unsafe customers. Workers knew these conditions made Covid‐19 transmission more likely and worried they were bringing Covid‐19 back to their loved ones. “Could I be carrying something?” asked a grocery store worker in MA. “Did I bring something home? We're not gonna know—everything takes two weeks,” referring to delays between Covid‐19 exposure, testing, and results at the pandemic's start.

#### 3.1.3 Uncertainty and Instability

Responding to dynamic Covid mitigation measures, the work that interviewees were allowed or required to do was repeatedly overhauled, month‐to‐month. This created profound uncertainty and instability (e.g., job loss or career change), which negatively affected their mental wellbeing. Workers discussed their experience managing such vulnerabilities, which compounded onto longstanding socioeconomic precarity—particularly for interviewees who identified as Black, African American, Hispanic/Latino/a, and/or women.

#### 3.1.4 Overburdening

Interviewees spoke about being overworked and overwhelmed. Because many workers in the food industry had been furloughed or laid off, those remaining on the job often had more work per person, plus new job duties like cleaning/sanitizing. Additional illness and exposure hazards that now accompanied normal job duties also weighed on workers. A grocery worker in NY summarized these burdens, saying, “I'm trying to stay safe, trying to get customers what they want. And it's really stressful for me, because I have to work double my number of shifts, since most of my colleagues were put out of the job. So, I had to replace them: I have to do my own job, and I do other people's jobs.”

Women and parents most often discussed overburdening, usually while describing increased caregiving work necessitated by the pandemic. For example, when a grocery worker in IL suddenly became the household's sole income, she had to take on all the hours she could, but being needed at home never stopped. “Sometimes,” she said, “I would hear my phone vibrating—the kids didn't know what to do for their homework, or the teacher was saying they're going to take points off. And it was really horrible.”

### 3.2 A Need for Self‐Reliance

Self‐reliance emerged from the interviews as a strong theme across food workers' expressions of stress and coping through the pandemic. Interviewees were quick to reorganize and make ends meet, explaining that support provided through employers was highly variable, fleeting, or delayed. “What is it like to work through the pandemic?” posed a grocery delivery driver in Illinois, “It's just—you just have to figure it out. There's no kind of plan; there's no one to tell you what to do. You're on your own trying to figure it out” (June 2, 2020). Alongside that necessity were interviewees' swift moves to control what they could control through repeated challenges. An Ohio‐based restaurant worker explained that process, saying:

“When it all started, I was getting really, really worried and stressed about the health of me and my family, and honestly the economy and the world…And then as I started going back to work and controlling what I could control, trying to be level‐headed and very careful, my stress (I guess inevitably, probably with the help of time, too) returned to somewhat normal.” (May 28, 2020)

The need for self‐reliance often took precedence over acknowledging personal distress, leading interviewees to downplay or suppress the extent to which pandemic‐related conditions affected them. A farmer in NY illustrated this, saying, “I'm not stressed, but I am worried about contracting the virus, and I work late, and there's no more money” (September 2, 2020).

## 4 Discussion

This qualitative study examined US food industry workers' experiences working through the 2020 Covid‐19 pandemic, finding that they described stress across 4 domains: financial strain, concern about getting/transmitting Covid‐19, sustained uncertainty, and overburdening. With only fleeting or delayed support from employers or state and federal agencies, workers relied on themselves to meet basic needs and manage ongoing challenges. These results align with similar studies on food industry workers' stress and health during Covid‐19, which reported elevated stress, food insecurity, and mental health strain, largely driven by economic precarity, job disruption, and unsafe working conditions (Josephson and Sanchez 2021; Loustaunau et al. 2021; Mayer et al. 2022; Spence et al. 2024). During the pandemic, fear of workplace exposure, inconsistent pay and hours, and lack of benefits such as paid sick leave were key contributors to occupational stress (Lippert et al. 2021; Mayer et al. 2022). These findings echoed pre‐pandemic accounts of chronic precarity, low wages, and inadequate protections in food work (Koenig et al. 2016), suggesting that pandemic‐related stressors intensified pre‐existing vulnerabilities rather than creating new conditions.

In other words, the Covid‐19 pandemic essentialized the already underpaid and undervalued work performed by 20 million people in the US food system—the nation's largest industry sector (Mai et al. 2023; Moore and Walle 2025; “US Bureau of Labor Statistics Industries at a Glance,” 2026). Subsequent research has documented the pandemic's disproportionate burden on food workers. A year‐long study by the United Food and Commercial Workers International Union found that essential workers were ten times more likely to have a Covid‐19 infection compared to the US population, and that their lives remain deeply affected by the pandemic (Michaud et al. 2022). In 2025, 52% of surveyed frontline grocery workers said their mental health suffered due to Covid‐19, with 44% reporting they were financially worse off, and 31% reporting physical health decline as a result of the pandemic (Schweizer 2026). These burdens were not evenly distributed. Black, Latinx, and immigrant workers were disproportionately concentrated in high‐risk essential roles with limited protections, reflecting deep labor inequities by race, ethnicity, gender, and immigration status (Corrado and Palumbo 2022; Allen et al. 2023; Haro‐Ramos et al. 2023; Jason et al. 2024). Qualitative research illustrated mechanisms that reinforce these inequities, as workers described how they needed the job, how this limited their ability to refuse risk, and how they were pressured to continue working under unsafe conditions (Loustaunau et al. 2021; Spence et al. 2024; Loustaunau 2026). Collectively, this research shows how the Covid‐19 pandemic was both an acute crisis and a syndemic context that compounded inequities in food work, amplifying chronic stress to adversely impact workers' health (Bogin and Varea 2020; Gravlee 2020; Kim 2024).

Exploitation and precarious conditions in food work have not stopped (Mayer et al. 2022; Blackstone et al. 2023; Wang and Churchill 2025). Recent research documents occupational risks that the pandemic accelerated or intensified, including the expansion of gig‐based food delivery work, conditions approximating forced labor, and pervasive wage theft (Blackstone et al. 2023; Moore and Walle 2025; Wang and Churchill 2025). Analysis of federal labor data up to 2024 finds that frontline food workers, who are primarily women of Color and immigrants, are 68% more likely to live in poverty compared to workers in other industries (Moore and Walle 2025). Across every sector of the food system, skilled labor required to grow, cook, butcher, prepare, deliver, and serve food has been systematically devalued along axes of race, gender, and citizenship (Minkoff‐Zern and Mares 2025). These and other recent studies demonstrate how precarious labor arrangements and worker exploitation are features, not failures, of a food system that depends on the disposability of its most vulnerable contributors. Post‐pandemic labor trends, including the “Great Resignation” and the Fight for $15 movement reflect collective refusal of the high‐risk, low‐wage labor that defines “essential” food system work (Minkoff‐Zern and Mares 2025). They also underscore momentum for enhanced worker protections (e.g., stronger labor rights, collective bargaining and living wages), reflecting broader grassroots organizing to improve conditions and secure workers' voices in their workplaces and communities (Anderson et al. 2021; Harriott 2024; Loustaunau 2026). As we continue to understand legacies of the Covid‐19 pandemic on human biology and stress physiology, it is vital that we center inequitable food work conditions and mobilize to transform them.

## 5 Conclusion

Interviews with food industry workers during the 2020 Coronavirus pandemic revealed overlapping stressors, namely: financial strain, infection risk, uncertainty, overburdening, and compelled self‐reliance. These findings reinforce prior evidence that food workers have long faced inadequate protections, a vulnerability intensified during the Covid‐19 pandemic. Strengthening structural support and incorporating food workers' lived experiences into research and policy are essential steps toward building a more equitable and resilient food system.

## Author Contributions

**Elizabeth Koselka:** conceptualization; funding acquisition; project administration; methodology; investigation; data curation; supervision; formal analysis; writing – original draft, review and editing.

## Funding

This project was supported by a LeCron Foster and Friends of Anthropology at Northwestern Research Grant from Northwestern University.

## Disclosure

AI was not used for this project, nor for writing and editing this manuscript.

## Ethics Statement

This study was reviewed and approved by the Institutional Review Board at Northwestern University.

## Conflicts of Interest

The author declares no conflicts of interest.

## Data Availability

The data that support this study's findings are available on request from the corresponding author. Data are not publicly available due to privacy or ethical restrictions.
